# Disproportionality analysis of drug-associated progressive multifocal leukoencephalopathy: roles of underlying diseases and immunomodulatory therapies in FAERS

**DOI:** 10.3389/fimmu.2025.1707211

**Published:** 2026-01-02

**Authors:** Xiaozhen Lin, Naishen Qin, Baoxia He, Jinhua Chen, Yajuan Zhang, Hui Wang, Weiling Liu

**Affiliations:** 1 Department of Pharmacy, The Affiliated Cancer Hospital of Zhengzhou University and Henan Cancer Hospital, HNHC Key Laboratory of Anti-tumor Drug Research (Henan Cancer Hospital), Henan Engineering Research Center for Tumor Precision Medicine and Comprehensive Evaluation, Zhengzhou, China; 2 School of Pharmacy, Guangxi Medical University, Nanning, Guangxi, China; 3 Department of Pharmacy, Xixia County People’s Hospital, Nanyang, China; 4 Department of Medical Oncology, The Affiliated Cancer Hospital of Zhengzhou University and Henan Cancer Hospital, Zhengzhou, China

**Keywords:** progressive multifocal leukoencephalopathy, pharmacovigilance, disproportionality analysis, FAERS, immunosuppressive therapy, monoclonal antibodies, time to onset

## Abstract

**Background:**

Progressive multifocal leukoencephalopathy (PML), a rare and often fatal JC virus–mediated disease, is a significant concern in immunocompromised patients.

**Objective:**

Following READUS-PV guidelines, this study evaluated disproportionality signals for PML associated with specific drugs and underlying diseases using the FDA Adverse Event Reporting System (FAERS).

**Methods:**

We identified PML cases in FAERS (2004 Q1–2024 Q4) and excluded those associated with HIV/AIDS. For drugs with ≥3 PML reports, disproportionality was assessed using the reporting odds ratio (ROR) and proportional reporting ratio (PRR), reported with 95% confidence intervals and χ² statistics, respectively. Subgroup analyses were conducted by age, sex, reporting region, and patient outcome. We also characterized the spectrum of underlying diseases and time to onset (TTO).

**Results:**

We analyzed 6,864 PML reports; in a sensitivity analysis excluding cases with TTO ≤60 days, 6,258 reports remained. Fifty-four drugs showed significant signals in primary analysis with the exception of acalabrutinib in the analysis restricted to 6,258 cases, including established high-risk agents and potential novel associations. Notably, we observed signals with four monoclonal antibodies (daratumumab, elotuzumab, epcoritamab, and isatuximab); isatuximab had no previous mentions in regulatory labels or published literature to our knowledge. Among established agents, natalizumab had the highest number of reports (n=1,848; ROR 40.7), and rituximab also showed a strong signal (n=1,296; ROR 41.8). PML was most frequently reported in multiple sclerosis (32.28%) and B-cell non-Hodgkin lymphomas (9.44%). TTO varied by agent; natalizumab showed the longest median TTO (44.0 months; 95% CI: 41.8–46.7). Median TTO for antineoplastic drugs (13.6 months; 95% CI: 11.5–15.9) was significantly shorter than for non-antineoplastic drugs (42.4 months; 95% CI: 39.7–44.1).

**Conclusions:**

These findings reinforce established and emerging PML reporting signals with immunomodulatory therapies and support heightened pharmacovigilance—particularly for novel monoclonal antibodies used in hematologic malignancies.

## 1 Introduction

Progressive multifocal leukoencephalopathy (PML) is a rare but often fatal demyelinating disease of the central nervous system (CNS). It is caused by human polyomavirus 2 (JCV), which establishes lifelong, persistent, and asymptomatic infection in most of the general population. CD4+ and CD8+ T-cell lymphopenia, resulting from HIV infection, chemotherapy, or immunosuppressive therapy are primary risk factors for PML (1). While acquired immune deficiency syndrome (AIDS) remains the most common global etiology of PML, modern immunosuppressive therapies, such as those for hematological malignancies, organ transplantation, and connective tissue diseases, can also induce iatrogenic PML (2, 3). Specifically, impaired cellular immunity may lead to JCV reactivation and subsequent development of PML. Historical evidence suggests that monoclonal antibody (mAb) immunotherapies, particularly natalizumab for multiple sclerosis (MS), have contributed to increased risk of iatrogenic PML (4, 5). The CD11α-targeting mAb efalizumab, previously used for psoriasis, was voluntarily withdrawn from the U.S. market in July 2009 because of the risk of PML (6). In addition, given case reports of PML with mAbs such as epcoritamab (for lymphoma) (7) and daratumumab (for multiple myeloma) (8–10), for which PML is not listed in their summary of product characteristics (SPC), therapy-induced immunosuppression warrants a comprehensive reassessment of PML risks (4).

PML is rare, and awareness among physicians, including neurologists and general radiologists, can be limited. Furthermore, there are no PML-specific symptoms (2, 11–13). Consequently, diagnosis is frequently delayed, with median symptom-to-diagnosis intervals ranging from 30 to 74 days across several studies; such delays may adversely affect prognosis (2, 11–13). To date, treatment options remain limited, and no therapeutic agent has demonstrated consistent efficacy against PML (14, 15). Withdrawal of immunosuppressive medications (or initiation of antiretroviral therapy in HIV-positive individuals) currently offers the only clinically validated survival benefit. However, when immune reconstitution cannot be promptly restored, prognosis is dismal. Notably, studies suggest that median survival following PML diagnosis is longer in HIV-infected than in HIV-uninfected patients (2). Moreover, the majority of PML survivors had no or only slight disability at follow-up, suggesting that earlier diagnosis may contribute to better outcomes (2). While Rindi’s review (4) found no PML signals for ocrelizumab, basiliximab, or tacrolimus under ideal conditions without additional immunosuppression (e.g., neoplasms, HIV, or concomitant immunomodulatory medications), real-world data indicate disease-dependent risks. Case reports describe PML in patients with MS following ocrelizumab treatment, despite no prior history of immunotherapy (16, 17).

With the continuing approval of novel mAbs and their expanding applications across autoimmune and lymphoproliferative disorders, the at-risk population has become increasingly heterogeneous. Despite therapeutic advances, progress in PML prevention has been limited, and many immunocompromised individuals remain at risk. We are therefore reappraising this subacute CNS inflammatory disease in non-HIV patients receiving drugs such as natalizumab, rituximab, and other recently approved agents. The FDA Adverse Event Reporting System (FAERS) is a spontaneous reporting database that collects adverse events (AEs) for FDA-approved drugs worldwide. Despite intrinsic limitations, it represents a valuable source of real-world data on drug safety profiles. It also allows estimation of time to onset (TTO) of adverse drug reactions (ADRs) (18), thereby informing prevention and improving the pharmacological management of iatrogenic disorders.

The objective of this study was to investigate reporting disproportionality for drug-associated PML using the FAERS database (19). The primary aim was to identify pharmaceuticals associated with PML reports. By elucidating these associations, we aim to emphasize the importance of recognizing potential PML-related risks and maintaining vigilant monitoring in clinical practice.

## 2 Methods

### 2.1 Data source

FAERS gathers spontaneous reports of AEs from health professionals, consumers, and manufacturers. The FAERS data files include seven datasets: patient demographic and administrative information (DEMO), drug information (DRUG), AEs (REAC), patient outcomes (OUTC), report sources (RPSR), therapy start and end dates for reported drugs, and reason for drug therapy (INDI) (20). Datasets were linked to each other via the case identifier (primary ID).

### 2.2 Data collection

American Standard Code for Information Interchange (ASCII) data packages from the first quarter of 2004 (2004Q1) to the fourth quarter of 2024 (2024Q4) were extracted from the FDA’s official website. If cases had the same case ID, the report with the most recent fda_date was retained; if both case ID and fda_date were identical, the report with the larger primary ID was retained (20).

AEs in REAC were coded by preferred terms (PTs) according to the Medical Dictionary for Regulatory Activities (MedDRA) Version 27.0. Reports with the PT “PML” (MedDRA code 10036807) were included for further analysis. Drugs associated with AEs were assigned roles (primary suspect, secondary suspect, concomitant, interaction); cases were included only if the drug was listed as the primary suspect for PML.

**Figure 1:**
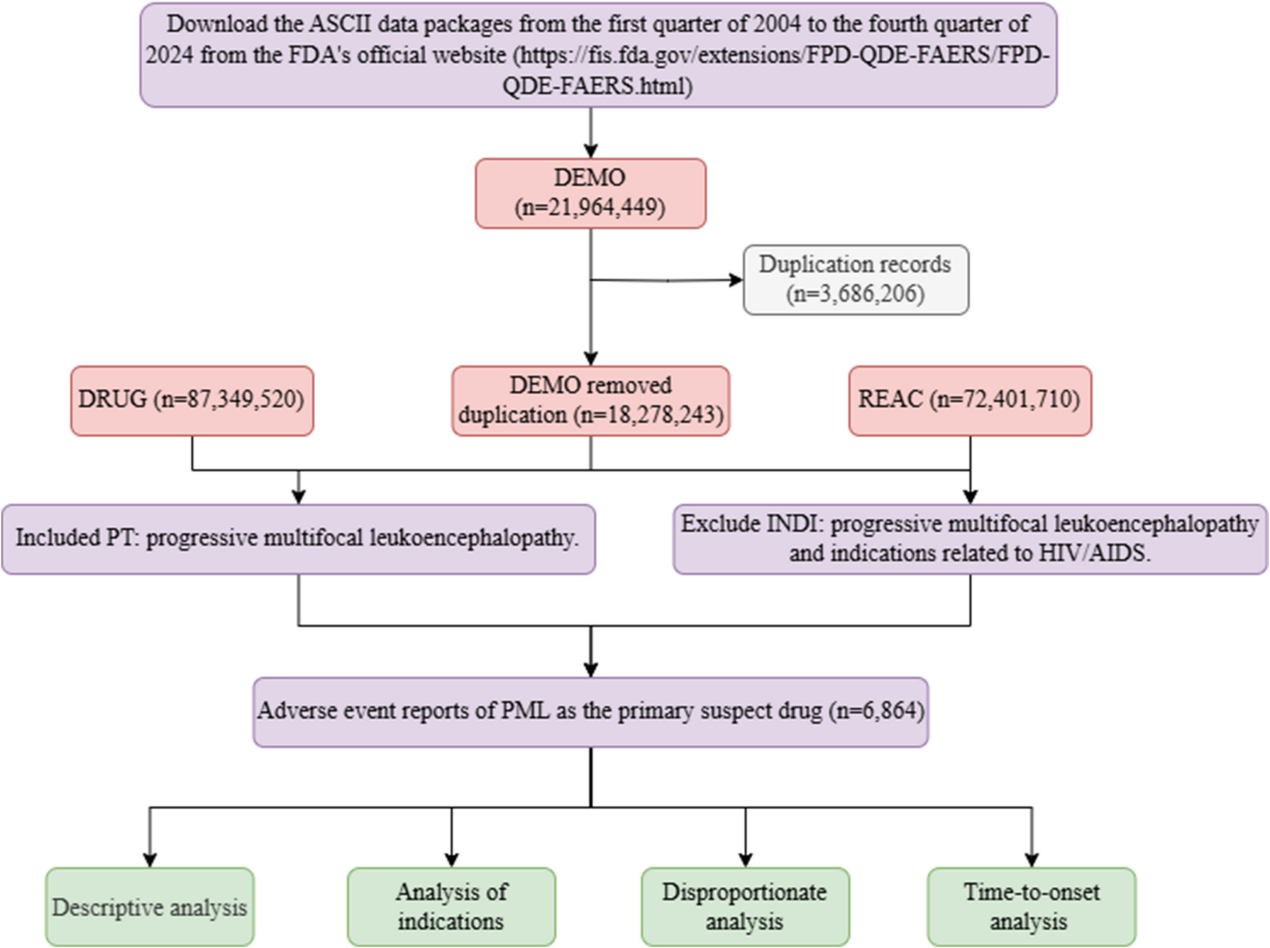
Flowchart of the study. *ASCII*, American Standard Code for Information Interchange; *PT*, Preferred Term, *FAERS*, FDA Adverse Event Reporting System; *MedDRA*, Medical Dictionary for Regulatory Activities; *DEMO*, patient demographic and administrative information, *DRUG*, drug information; *REAC*, adverse events; *INDI*, Reason for drug therapy; *PML*, progressive multifocal leukoencephalopathy.

To avoid the potential impact of HIV infection or AIDS on results due to immunodeficiency, we excluded cases with INDI entries indicating “HIV” or “AIDS” (including “Prophylaxis against HIV infection” or “Antiretroviral therapy”). We also excluded cases with INDI explicitly indicating “PML” and removed records with an onset date earlier than the medication start date. To ensure methodological transparency and reproducibility, this study adhered to the READUS-PV guideline (21, 22). The flow of data processing is detailed in **Figure 1**.

### 2.3 Statistical analysis

Descriptive analyses summarized clinical profiles of patients with drug-associated PML, including age, sex, INDI, occupation, outcome, and reporting country. Signals of potential associations between PML and suspected drugs were evaluated using the reporting odds ratio (ROR) (23) and the proportional reporting ratio (PRR) (24). A drug-AE signal was considered positive only when both algorithms met their respective thresholds; the formulas and thresholds are provided in **Table 1**.

**Table 1:** Two algorithms used for signal detection.

Algorithms	Calculation formula	Criteria
ROR	ROR=(a/c)/(b/d)	a≥3, 95%CI>1
	$95\%CI=e^{In(ROR)\pm 1.96{\left(\frac{1}{a}+\frac{1}{b}+\frac{1}{c}+\frac{1}{d}\right)}^{0.5}}$	
PRR	PRR=[a/(a + b)]/[c/ (c + d)]	a≥3, PRR≥2, χ^2^≥4
	$\chi^{2}=[(ad-bc{)}^{2}(a\;+\;b\;+\;c\;+\;d)/[(a\;+\;b)(c\;+\;d)(a\;+\;c)(b\;+\;d)]$	

*a* number of reports of the suspected drug with the suspected ADR, *b* number of reports of the suspected drug without the suspected ADR, *c* number of reports of other drugs with the suspected ADR, *d* number of reports of other drugs without the suspected ADR, *95% CI* 95% confidence interval, *N* the number of reports, χ^2^ chi-squared

Because PML occurring within a very short period after drug initiation is unlikely to be attributable to the drug—typically manifesting weeks to months after immunosuppression (4, 14); cases with TTO ≤60 days were excluded from further ROR/PRR analyses. TTO was defined as the interval between the initiation date of the suspect drug (as recorded in Individual Case Safety Reports) and the first documented occurrence of PML. Cases with incomplete date information were excluded from TTO analyses. We compared the cumulative incidence of antineoplastic drugs versus non-antineoplastic drugs. The 95% confidence interval (CI) for the median TTO was estimated using nonparametric bootstrapping with 1,000 replicates. Data mining and statistical analyses were performed using GraphPad Prism 9.5.1 and R 4.4.2 (R Core Team).

## 3 Results

### 3.1 Baseline characteristics of PML

**Figure 2:**
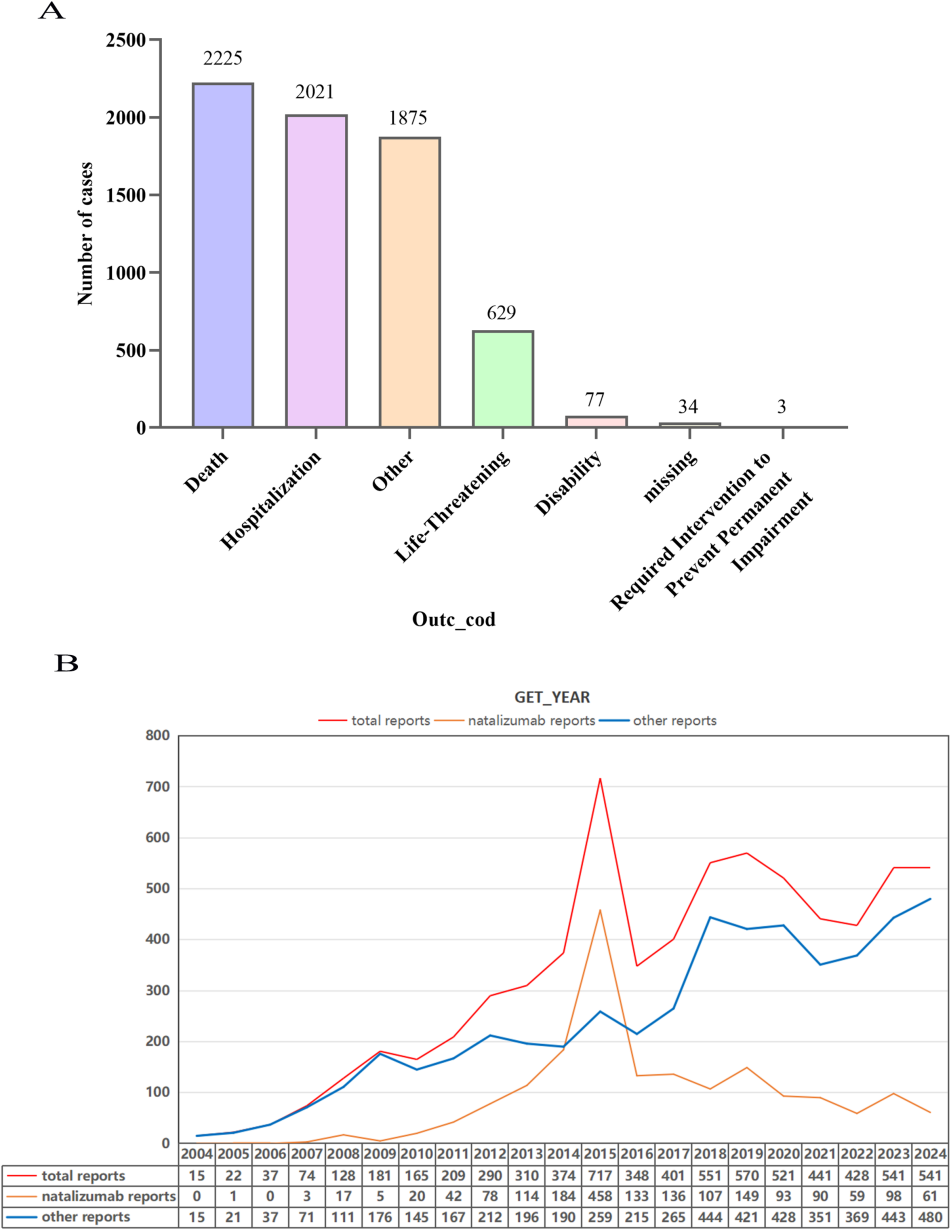
**(A)** Outcomes of drug-related PML reports in FAERS, 2004Q1–2024Q4. **(B)** Temporal trends of drug-related PML reports in FAERS, 2004Q1–2024Q4.

The baseline characteristics of drug-associated PML are shown in **Table 2** and **Figure 2**. A total of 6,864 reports of PML were identified. Most reports (73.02%) involved serious outcomes, including death, hospitalization, life-threatening events, disability, and interventions to prevent permanent impairment. Death was the most frequent serious outcome (2,225; 32.4%), followed by hospitalization (2,021; 29.4%), life-threatening (629; 9.2%), disability (77; 1.1%), and interventions to prevent permanent impairment (3; 0.04%). Overall, drug-associated PML reports increased over time, with a notable peak in 2015, a slight decline thereafter, and then a resumption of the general upward trend. Natalizumab-associated PML showed a similar peak in 2015 followed by a modest decline, whereas non-natalizumab PML reports rose steadily from 2004 to 2024. Baseline characteristics after excluding cases with TTO ≤60 days are shown in **Supplementary Table 1**.

**Table 2:** Baseline characteristics of included FAERS reports (2004Q1–2024Q4).

Characteristics	Number of reports, N (%)
Sex	
Female	3,654 (53.2%)
Male	2,559 (37.3%)
Missing	651 (9.5%)
Age	
<18	88 (1.3%)
18-44	1,310 (19.1%)
45-59	1,844 (26.9%)
≥60	2,118 (30.9%)
Missing	1,504 (21.9%)
Reporter Occupation	
Doctor of Medicine	3,548 (51.7%)
Healthcare Professional	1,058 (15.4%)
Pharmacist	183 (2.7%)
Registered Nurse	1 (0.001%)
Other health professional	1,258 (18.3%)
Consumer	612 (8.9%)
Lawyer	11 (0.2%)
Missing	193 (2.8%)
Reporter Country	
United States	1,864 (27.2%)
Germany	664 (9.7%)
France	590 (8.6%)
United Kingdom	529 (7.7%)
Japan	422 (6.1%)
Others	2,795 (40.7%)

### 3.2 Reason for drug therapy analysis of drug-related PML reports

**Figure 3:**
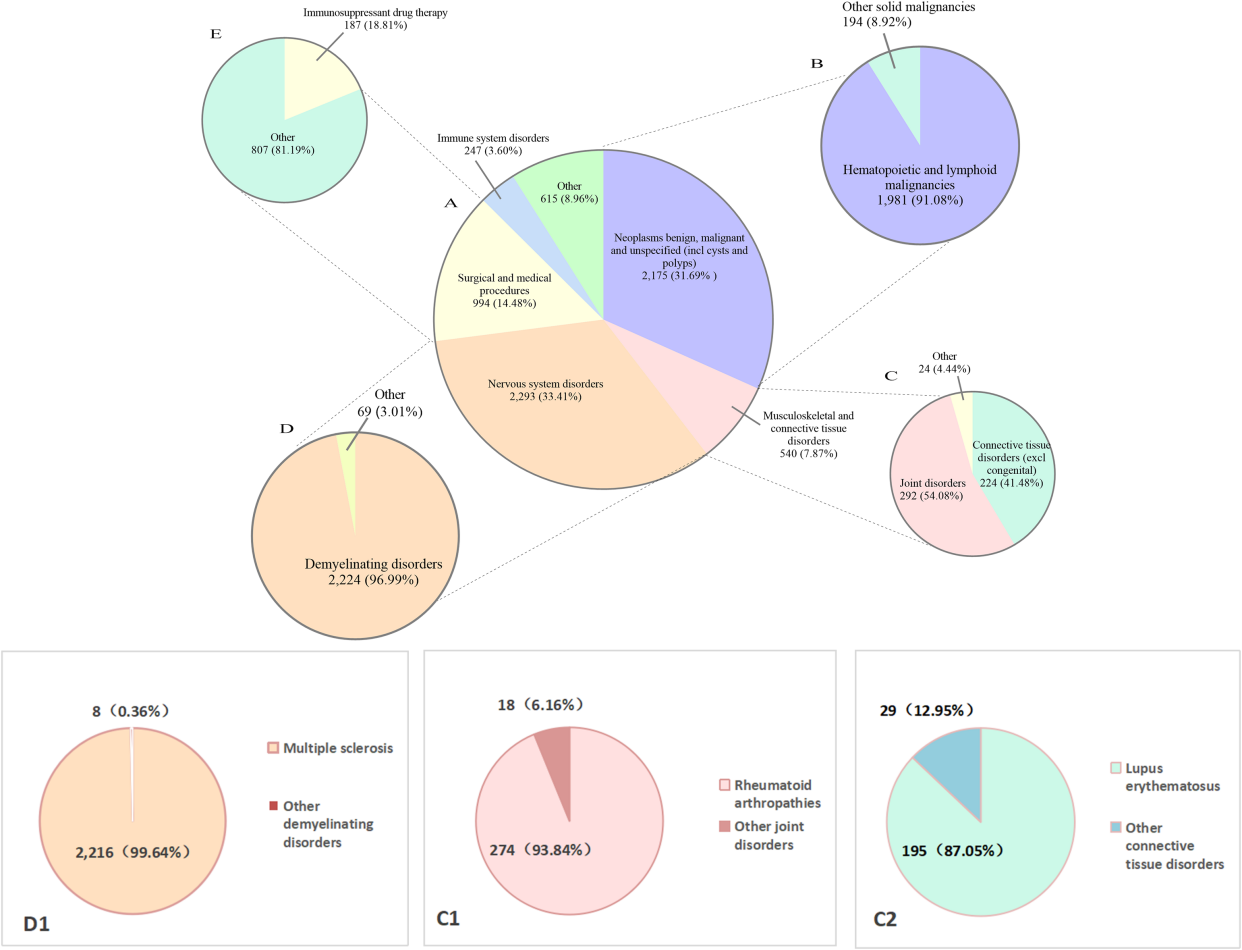
Proportional distribution of drug-related PML reports by clinical reason for drug therapy, FAERS (2004Q1-2024Q4). D1: Demyelinating disorders, C1: Joint disorders, C2: Connective tissue disorders (excl. congenital).

The major INDI categories among PML-related reports are presented in **Table 3**. We further summarized INDIs using broad System Organ Class groupings (**Figure 3**) and provided a detailed sub-classification of hematopoietic and lymphoid malignancies. In this sub-classification, 1,084 cases (15.79%) were lymphomas, as detailed in **Table 4**. The distribution of INDIs after excluding cases with TTO ≤60 days is provided in **Supplementary Table 2**, **3** and **Supplementary Figure**.

**Table 3:** Reported drug-related PML case profiles: Clinical reason for drug therapy from 2004Q1 to 2024Q4.

Reason for drug therapy	System organ class	N (%)
Multiple sclerosis acute and progressive	Nervous system disorders	2,216 (32.28%)
Hematopoietic and lymphoid malignancies	Neoplasms benign, malignant and unspecified (incl. cysts and polyps)	1,981 (28.86%)
Rheumatoid arthropathies	Musculoskeletal and connective tissue disorders	274 (3.99%)
Immune system disorders	Immune system disorders	247 (3.60%)
Lupus erythematosus (incl. subtypes, LE)	Musculoskeletal and connective tissue disorders	195 (2.84%)
Other solid malignancies	Neoplasms benign, malignant and unspecified (incl. cysts and polyps)	194 (2.83%)
Immunosuppressant drug therapy	Surgical and medical procedures	187 (2.72%)
Other reason for drug therapy	*	1,570 (22.87%)

*incl.* Including. * involving multiple system organ classes or with missing data.

**Table 4:** Sub-classification of hematopoietic and lymphoid malignancies among drug-related PML cases: Clinical reason for drug therapy (2004Q1-2024Q4).

Hematopoietic and lymphoid malignancies	Reports N (%)
Lymphomas non-Hodgkin’s B-cell	648 (9.44%)
Lymphomas non-Hodgkin’s T-cell	30 (0.44%)
Lymphomas non-Hodgkin’s unspecified histology	258 (3.76%)
Lymphomas Hodgkin’s disease	65 (0.95%)
Lymphomas unspecified NEC	83 (1.21%)
Leukemias	564 (8.22%)
Plasma-cell neoplasms	298 (4.34%)
Hematopoietic neoplasms (excl leukaemias and lymphomas)	35 (0.51%)
Total	1,981 (28.86%)

*NEC* Not Elsewhere Classifiable, *excl* excluding.

### 3.3 Disproportionality analysis

In the full dataset (6,864 cases), 57 drugs met both disproportionality criteria (a≥3; lower limit of the 95% CI for ROR >1; PRR ≥2; χ² ≥4), including three antiretroviral agents—abacavir/dolutegravir/lamivudine, stavudine, and zidovudine. For these three drugs, all reports lacked a documented reason for therapy; given that antiretroviral treatment reduces the risk of HIV-related PML (14), these agents were excluded from further analysis. In the analysis restricted to 6,258 cases (after excluding the three drugs), 53 drugs continued to meet the same criteria with the exception of acalabrutinib. **Table 5** lists their pharmacological classes along with ROR (95% CI), PRR, and χ² values. Drug classes included antineoplastic drugs (31/54), immunosuppressants (17/54), glucocorticoids (4/54), an antimalarial (1/54), and a chimeric antigen receptor T-cell therapy (1/54).

**Table 5:** Classification of 54 PML-associated drugs by primary indication and disproportionality analysis (ROR and PRR) based on FAERS (2004Q1-2024Q4).

Drug classifications		Substance	N	Total 6,864 cases		Total 6,258 cases (TTO > 60 days or missing)			PML acknowledged in FDA SPC?
				ROR (95%CI)	PRR (X^2^)	N	ROR (95%CI)	PRR (X^2^)	
Antineoplastic drugs	Cytotoxic drugs	methotrexate	186	3.95 (3.41–4.57)	3.94 (397.56)	180	4.20 (3.62–4.87)	4.19 (425.06)	No**^#^**
		doxorubicin	132	11.21 (9.43–13.32)	11.16 (1,198.39)	113	10.51 (8.72–12.66)	10.48 (951.35)	No
		cyclophosphamide	120	11.06 (9.23–13.25)	11.02 (1,074.03)	105	10.60 (8.74–12.86)	10.57 (894.74)	No
		bendamustine	101	29.42 (24.14–35.84)	29.11 (2,702.54)	89	28.42 (23.03–35.06)	28.16 (2,298.93)	Yes
		fludarabine	84	29 (23.36–36.00)	28.70 (2,219.34)	76	28.77 (22.92–36.12)	28.51 (1,993.56)	Yes
		busulfan	35	15.39 (11.03–21.47)	15.31 (465.89)	35	16.89 (12.10–23.57)	16.80 (517.33)	No
		etoposide	27	6.16 (4.22–8.99)	6.15 (116.01)	23	5.76 (3.82–8.67)	5.75 (89.86)	No
		vincristine	24	8.67 (5.80–12.95)	8.64 (161.69)	23	9.11 (6.05–13.73)	9.09 (164.98)	No
		melphalan	24	10.11 (6.77–15.10)	10.07 (195.54)	23	10.63 (7.05–16.01)	10.59 (199.12)	No
		azacitidine	19	3.14 (2.00–4.93)	3.14 (27.61)	19	3.45 (2.20–5.41)	3.44 (32.84)	No
		chlorambucil	16	42.39 (25.86–69.50)	41.75 (635.15)	16	46.51 (28.36–76.25)	45.8 (699.64)	No
		epirubicin	10	4.12 (2.21–7.66)	4.11 (23.51)	8	3.61 (1.80–7.23)	3.61 (15.06)	No
		thiotepa	8	14.43 (7.20–28.92)	14.36 (99.34)	8	15.83 (7.90–31.72)	15.75 (110.40)	No
		mitoxantrone	6	8.89 (3.99–19.81)	8.86 (41.82)	5	8.12 (3.38–19.54)	8.10 (31.12)	No
		hydroxycarbamide	4	2.80 (1.05–7.47)	2.80 (4.63)	4	3.07 (1.15–8.20)	3.07 (5.59)	No
		idarubicin	4	9.44 (3.54–25.2)	9.41 (30.06)	4	10.35 (3.88–27.64)	10.32 (33.66)	No
	Novel antineoplastic drugs	rituximab*	1,296	41.80 (39.34–44.42)	41.29 (41345.77)	1,217	43.35 (40.71–46.17)	42.86 (40,090.06)	Yes
		bortezomib	51	4.28 (3.25–5.64)	4.28 (127.05)	45	4.14 (3.09–5.55)	4.14 (106.31)	Yes
		brentuximab vedotin*	47	17.9 (13.42–23.87)	17.79 (739.98)	40	16.70 (12.23–22.81)	16.62 (583.47)	Yes
		obinutuzumab*	42	14.75 (10.88–19.99)	14.68 (532.15)	39	15.02 (10.96–20.60)	14.95 (504.81)	Yes
		daratumumab	33	7.51 (5.33–10.58)	7.50 (184.93)	29	7.24 (5.03–10.43)	7.23 (154.89)	No
		ofatumumab	30	3.06 (2.13–4.38)	3.05 (41.28)	21	2.34 (1.53–3.60)	2.34 (16.12)	Yes
		carfilzomib	22	4.38 (2.88–6.66)	4.38 (57.13)	21	4.59 (2.99–7.04)	4.58 (58.63)	Yes
		idelalisib	15	7.10 (4.28–11.79)	7.08 (78.24)	13	6.75 (3.91–11.64)	6.74 (63.39)	No
		ibritumomab tiuxetan	14	41.71 (24.59–70.74)	41.09 (546.64)	14	45.75 (26.97–77.61)	45.07 (602.18)	No
		polatuzumab vedotin	9	10.49 (5.45–20.19)	10.45 (76.86)	7	8.95 (4.26–18.79)	8.92 (49.20)	Yes
		teclistamab	7	10.78 (5.13–22.66)	10.75 (61.83)	7	11.83 (5.63–24.86)	11.79 (69.05)	Yes
		acalabrutinib	7	2.55 (1.22–5.36)	2.55 (6.60)	4	1.60 (0.60–4.26)	2.00 (0.90)	Yes
		elotuzumab	5	7.78 (3.23–18.72)	7.76 (29.44)	5	8.54 (3.55–20.54)	8.51 (33.14)	No
		epcoritamab	4	11.15 (4.17–29.77)	11.11 (36.78)	3	9.17 (2.95–28.49)	9.14 (21.76)	No
		isatuximab	3	4.12 (1.33–12.78)	4.11 (7.07)	3	4.52 (1.46–14.02)	4.51 (8.20)	No
Immuno–suppressant drugs	Antimetabolite drugs	mycophenolate mofetil*	271	12.75 (11.29–14.40)	12.70 (2,805.68)	255	13.18 (11.62–14.94)	13.12 (2740.48)	Yes
		azathioprine*	32	14.65 (10.34–20.75)	14.57 (402.85)	32	16.07 (11.35–22.77)	15.99 (447.63)	Yes
		leflunomide	18	3.13 (1.97–4.97)	3.13 (26.00)	18	3.43 (2.16–5.46)	3.43 (30.94)	No
		cladribine	10	4.67 (2.51–8.69)	4.66 (28.74)	9	4.61 (2.40–8.87)	4.60 (25.36)	Yes
	Calcineurin inhibitors	tacrolimus*	133	6.12 (5.15–7.26)	6.11 (557.14)	126	6.36 (5.33–7.59)	6.35 (556.78)	Yes
		cyclosporine	89	4.87 (3.95–6.01)	4.87 (269.87)	84	5.05 (4.07–6.26)	5.04 (268.42)	Yes
	Antiproliferative agents	sirolimus	9	2.86 (1.49–5.50)	2.86 (10.84)	9	3.13 (1.63–6.03)	3.13 (13.05)	No
	Other immunosuppressants	fingolimod	245	8.28 (7.29–9.41)	8.26 (1,507.19)	232	8.61 (7.55–9.82)	8.59 (1,498.36)	Yes
		dimethyl fumarate	87	2.19 (1.77–2.70)	2.19 (55.40)	83	2.29 (1.85–2.85)	2.29 (59.57)	Yes
		belatacept*	16	17.22 (10.52–28.16)	17.11 (242.26)	16	18.89 (11.55–30.90)	18.77 (268.62)	Yes
		antithymocyte immunoglobulin	8	3.80 (1.90–7.60)	3.79 (16.43)	6	3.12 (1.40–6.95)	3.12 (8.63)	No
		natalizumab*	1,848	40.70(38.58–42.94)	40.26 (51,725.03)	1,670	40.21 (38.01–42.54)	39.82 (46,351.35)	Yes
		ocrelizumab	57	3.05 (2.35–3.96)	3.05 (77.98)	46	2.70 (2.02–3.61)	2.70 (48.84)	Yes
		alemtuzumab	44	10.62 (7.89–14.29)	10.58 (379.32)	42	11.12 (8.20–15.07)	11.08 (382.73)	Yes
		belimumab	23	2.66 (1.77–4.01)	2.66 (23.81)	21	2.67 (1.74–4.10)	2.67 (21.81)	Yes
		efalizumab	13	13.56 (7.86–23.40)	13.50 (150.19)	13	14.88 (8.62–25.67)	14.81 (167.06)	Yes
		basiliximab	5	8.21 (3.41–19.75)	8.19 (31.53)	5	9.00 (3.74–21.67)	8.98 (35.43)	Yes
Glucocorticoid		prednisolone	143	11.79 (9.98–13.91)	11.74 (1,376.17)	137	12.40 (10.46–14.69)	12.35 (1,398.39)	No
		prednisone	113	8.79 (7.30–10.59)	8.76 (764.65)	112	9.57 (7.93–11.54)	9.54 (841.27)	No
		dexamethasone	88	5.72 (4.63–7.06)	5.71 (337.63)	88	6.28 (5.09–7.75)	6.27 (384.50)	No
		methylprednisolone	74	8.03 (6.38–10.10)	8.01 (448.91)	73	8.69 (6.90–10.95)	8.67 (489.69)	No
Antimalarial drugs		hydroxychloroquine	51	8.59 (6.52–11.32)	8.57 (338.63)	51	9.43 (7.16–12.43)	9.41 (380.17)	No
CAR-T drugs		axicabtagene ciloleucel	18	7.27 (4.57–11.55)	7.25 (96.83)	16	7.09 (4.34–11.58)	7.07 (83.25)	No

*CI* confidence interval, *ROR* reporting odds ratio, *PRR* proportional reporting ratio, *CAR-T drugs* chimeric antigen receptor T-cell immunotherapy drugs, *SPC* the summary of product characteristics, * carry boxed warnings of PML, ***#*** PML acknowledged in the European Medicines Agency’s SPC

The top 10 drugs by report frequency were natalizumab (1,848; 26.92%), rituximab (1,296; 18.88%), mycophenolate mofetil (271; 3.95%), fingolimod (245; 3.57%), methotrexate (186; 2.71%), prednisolone (143; 2.08%), tacrolimus (133; 1.94%), doxorubicin (132; 1.92%), cyclophosphamide (120; 1.75%), and prednisone (113; 1.64%).

The top 10 drugs by signal intensity (ROR) were chlorambucil (ROR 42.39; PRR 41.75), rituximab (ROR 41.8; PRR 41.29), ibritumomab tiuxetan (ROR 41.71; PRR 41.09), natalizumab (ROR 40.7; PRR 40.26), bendamustine (ROR 29.42; PRR 29.11), fludarabine (ROR 29.00; PRR 28.70), brentuximab vedotin (ROR 17.90; PRR 17.79), belatacept (ROR 17.22; PRR 17.11), busulfan (ROR 15.39; PRR 15.31), and obinutuzumab (ROR 14.75; PRR 14.68). Rank ordering by ROR changed slightly across the two analyses (see **Supplementary Table 4**).

### 3.4 The TTO of drug-related PML

**Figure 4:**
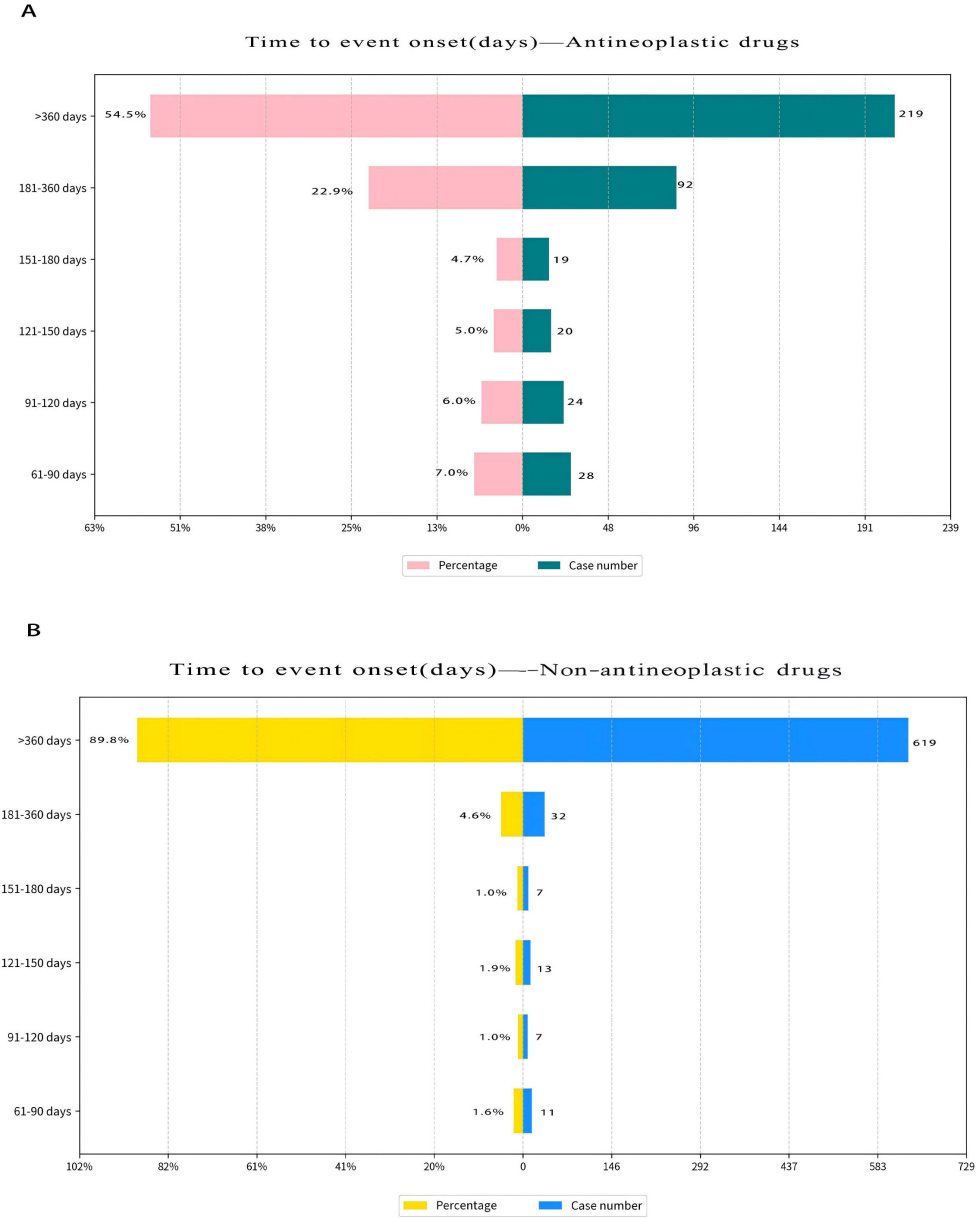
**(A)** Time to event onset for antineoplastic drugs. **(B)** Time to event onset for non-antineoplastic drugs.

**Figure 5:**
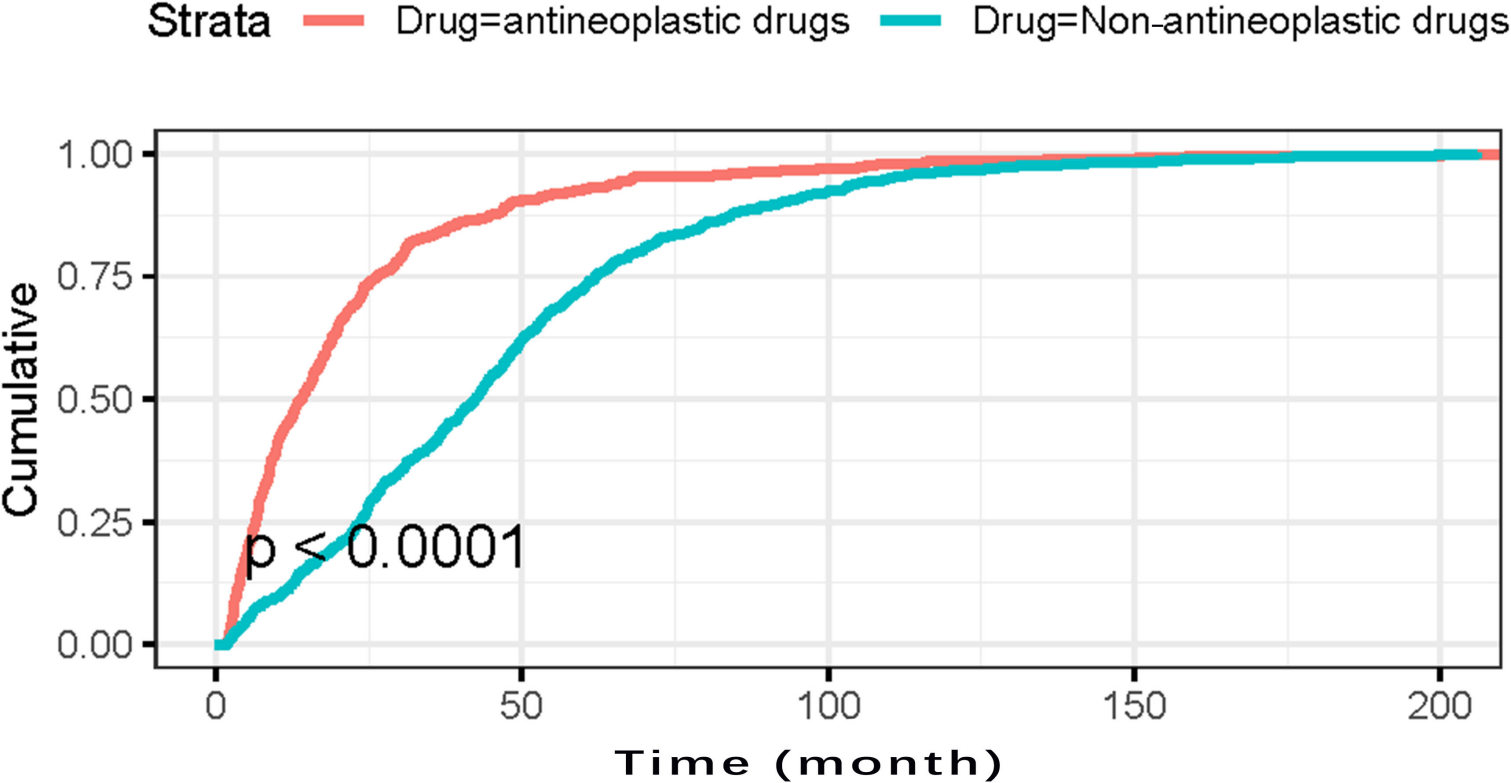
Cumulative incidence rate of drug-related PML for antineoplastic drugs and non-antineoplastic drugs after exclusion of cases with TTO ≤ 60 days over 200 months (log-rank test).

After excluding records with incomplete dates and cases with TTO ≤60 days, 1,091 reports across the 54 PML-associated drugs remained. The TTO distribution had a median of 29.2 months (95% CI: 26.4–31.0); 76.81% (n=838) occurred after the first 360 days following drug initiation (**Figure 4**). Summary statistics for drugs with ≥10 retained cases are shown in **Table 6**. We provide the median and interquartile range (IQR) of raw TTO data for drugs with 5 to 9 valid cases (i.e., >5 and ≤10) in **Supplementary Table 6**. Log-rank tests indicated significant differences between antineoplastic and non-antineoplastic drug groups in TTO distributions (*P<0.0001*) (**Figure 5**).

**Table 6:** Time to onset analysis of progressive multifocal leukoencephalopathy for top 10 implicated drugs (2004Q1-2024Q4).

Drug name	N	Interquartile range (m)	Median (95% CI) (m)	Minimum (m)	Maximum (m)
natalizumab	580	26.2–63.8	43.9 (42.0–46.6)	2.0	201.0
rituximab	276	6.5–26.5	14.1 (11.0–17.0)	2.0	216.0
fingolimod	43	13.0–65.6	43.0 (24.4–54.1)	2.1	206.0
cyclophosphamide	26	5.0–23.3	15.8 (9.0–18.0)	2.4	84.0
mycophenolate mofetil	22	10.3–55.8	25.7 (12.1–50.0)	2.0	113.7
fludarabine	15	7.0–24.0	10.0 (7.0–24.0)	3.1	116.0
tacrolimus	14	9.5–55.7	17.4 (11.0–49.0)	3.9	71.9
obinutuzumab	14	5.5–20.5	13.9 (6.6–20.3)	2.2	23.1
dimethyl fumarate	13	4.2–54.4	22.1 (5.5–54.1)	2.0	74.6
bendamustine	13	6.1–17.0	10.2 (6.2–14.0)	2.8	29.9
antineoplastic drugs	402	6.5–25.6	13.6 (11.5–15.9)	2.0	206.0
non–antineoplatic drugs	689	23.8–62.0	42.4 (39.7–44.1)	2.0	216.0
>60 days	1,091	12.8–52.9	29.2 (26.4–31.0)	2.0	216.0

*N* number, *m* month, one year = 365.25 days.

## 4 Discussion

PML is a life-threatening complication. Drugs are an important etiology, notably natalizumab (25–28) and rituximab (1, 29, 30). Despite the expanded clinical use of novel agents—particularly mAbs—no large, comprehensive FAERS case analyses have been reported in the past decade. To our knowledge, two prior studies used ROR-based signal detection for PML: Schmedt et al. (2012) (31) examined immunosuppressant-associated PML in FAERS with thresholds of ≥3 cases and ROR >2 with 95% CI >1, and Melis et al. (2015) (32) evaluated drug-related PML and leukoencephalopathy in VigiBase using the same criteria. Both excluded patients with HIV/AIDS; additionally, Melis et al. excluded cases with myelo- or lymphoproliferative disorders, rheumatoid arthritis, or systemic LE. In our study, we excluded HIV/AIDS-related indications (including “Prophylaxis against HIV infection” and “Antiretroviral therapy”), enabling a more specific assessment of drug-associated PML signals in real-world populations while limiting confounding from antiretroviral use.

Although PML typically manifests weeks to months after immunosuppression, cases with TTO <60 days are likely influenced by other risk factors or synergistic effects. However, over half of reports lacked either an event date or a drug start date; excluding all incomplete records would cause substantial information loss. We therefore compared TTO between antineoplastic and non-antineoplastic drugs and, for TTO estimation, applied stricter inclusion by excluding cases with TTO ≤60 days. One hypothesis is that the shorter TTO observed with antineoplastic agents reflects more profound immunosuppression than with other drugs, even accounting for disease characteristics such as myelo- or lymphoproliferative disorders (33).

We observed more PML reports among females than males, which may partly relate to the higher prevalence of autoimmune diseases (e.g., MS, systemic LE) in females and consequent exposure to immunomodulatory/immunosuppressive therapies. Natalizumab-related PML reports showed an upward trend with a peak in 2015, followed by a modest decline, whereas non-natalizumab PML reports increased steadily from 2004 to 2024. In 2015, natalizumab-related reports reached 458, exceeding recent years. TOUCH^®^ Risk Evaluation and Mitigation Strategies (REMS) for natalizumab were initially approved on 11/7/2011, with significant modifications on 12/15/2013 and 5/12/2015 (34). Notably, among natalizumab-related reports in 2015, 189 events occurred before 2014 (**Supplementary Table 5**). The decline after 2015 may reflect the broad adoption of TOUCH^®^ REMS. Consistent patterns have been reported elsewhere: in France, natalizumab-related PML incidence increased by 45.3% annually before 2013 but decreased by 23.0% per year from 2013 to 2016 (26), and in Sweden, cumulative PML incidence fell from 1.49 per 1000 person-years in 2012 to 0.72 in 2018 (35). The rise in non-natalizumab PML reports may be linked to the introduction of new mAbs such as Brentuximab vedotin (FDA-approved 08/19/2011; boxed warning for PML), Obinutuzumab (FDA-approved 11/01/2013; boxed warning for PML), and Ocrelizumab (FDA-approved 03/28/2017; warning for PML).

In the initial analysis, 57 drugs met both disproportionality criteria; after excluding abacavir/dolutegravir/lamivudine, stavudine, and zidovudine, 54 drugs remained. For abacavir/dolutegravir/lamivudine (indication “HIV infection, treatment”), all three PML reports lacked a recorded reason for therapy; residual immunosuppression from HIV infection may have contributed. Likewise, stavudine and zidovudine each had three PML reports without documented indications. Because antiretroviral therapy reduces HIV-related PML risk (2, 14), and these nucleoside reverse transcriptase inhibitors are used in combination regimens unlikely to directly trigger JCV reactivation (14), we excluded these drugs from further analyses. Acalabrutinib, a highly selective, potent, covalent Bruton tyrosine kinase inhibitor, was first approved by FDA in 2017 with PML warnings for adult patients with relapsed/refractory mantle cell lymphoma. Its indications were subsequently expanded to include (1): combination with bendamustine and rituximab for the treatment of adult patients with previously untreated mantle cell lymphoma who are ineligible for autologous hematopoietic stem cell transplantation (ASCT); and (2) the treatment of adult patients with chronic lymphocytic leukemia (CLL) or small lymphocytic lymphoma. It is important to note that although acalabrutinib carries PML warnings in its SPC, PML cases have only been observed during the clinical trial phase (36). No PML signal was identified for this agent after excluding cases with TTO ≤60days—only 4 PML reports remained.

Of the 54 included drugs, 28 did not carry a labeled warning. We identified novel, disproportionality signals for four mAbs: daratumumab, elotuzumab, epcoritamab, and isatuximab, of which isatuximab had no previous reports in regulatory documents or the literature to our knowledge. Of these, daratumumab, elotuzumab, and isatuximab are indicated for multiple myeloma (MM), and daratumumab/isatuximab are anti-CD38 mAbs. We also confirmed TTO associations for 10 drugs: natalizumab, rituximab, fingolimod, cyclophosphamide, mycophenolate mofetil (MMF), fludarabine, tacrolimus, obinutuzumab, dimethyl fumarate, and bendamustine.

Daratumumab, the first human anti-CD38 mAb approved by the FDA for MM in 2015, is now widely used. Although PML is not recognized in its SPC, several case reports have been published (8, 10, 37–39). Three reported cases occurred in patients with MM who had received multiple prior lines of therapy. A 63-year-old man, status post ASCT, developed PML while on pomalidomide maintenance; his prior therapies included lenalidomide, carfilzomib, daratumumab, and dexamethasone. He died within weeks of the PML diagnosis (8). A 63-year-old woman with relapsed/refractory MM receiving daratumumab and pomalidomide developed PML after seven years of disease, multiple prior treatments (including high-dose melphalan and ASCT), and hypogammaglobulinemia (9). Steinhardt et al. described PML in a 59-year-old man with an 11-year MM history and seven prior therapy lines (including allogeneic transplantation 6.5 years earlier) after 20 cycles of daratumumab, pomalidomide, bortezomib, cyclophosphamide, and dexamethasone (10). Isatuximab, a newer anti-CD38 mAb, was approved by the FDA and EMA in 2020 for MM. While no isatuximab-associated PML cases have appeared in the peer-reviewed literature, three cases were identified in FAERS, all in plasma-cell myeloma. Increased vigilance is warranted as clinical use broadens.

Elotuzumab, approved in 2015, is a SLAMF7-directed immunostimulatory antibody indicated in combination with lenalidomide (or pomalidomide) and dexamethasone for MM. One PML case has been reported in a 73-year-old patient with BJP-κ MM and mild lymphocytopenia (40), suggesting that lymphocytopenia may be an additional predisposing factor.

Epcoritamab, a bispecific CD20-directed CD3 T-cell engager approved in 2023 for refractory diffuse large B-cell lymphoma (DLBCL), has also been linked to PML. Lisecki et al. reported a woman with a 9-year history of follicular lymphoma who developed PML after the 11th dose and died 3 months later (7). In our analysis, four cases of epcoritamab-associated PML were found in FAERS: one in DLBCL and three in follicular lymphoma.

Idelalisib (FDA-approved 2014), a PI3Kδ inhibitor indicated for relapsed CLL (with rituximab), follicular B-cell NHL, and small lymphocytic lymphoma, has been associated with PML-immune reconstitution inflammatory syndrome in a CLL patient after multiple prior treatments and ASCT (41). A pharmacovigilance signal has also been reported (42).

For the drugs mentioned above, it remains challenging to determine whether the observed PML cases are ascribed to the drug itself or to the underlying hematologic malignancy. Zaheer and Berger classified drugs associated with PML into 3 classes (43). These drugs have a mechanism of action that may suggest a potential for increased PML risk and/or with which rare cases of PML have been observed (43).

The ROR and PRR values in **Table 5** quantify the strength of association between PML reporting and the enrolled drugs. The top ten drugs by ROR were chlorambucil, rituximab, ibritumomab tiuxetan, natalizumab, bendamustine, fludarabine, brentuximab vedotin, belatacept, busulfan, and obinutuzumab. Five high-signal drugs carry boxed warnings for PML (rituximab, natalizumab, brentuximab vedotin, belatacept, and obinutuzumab). Three drugs, chlorambucil, ibritumomab tiuxetan, and busulfan, showed high disproportionality in our study but do not list PML in their SPCs. Chlorambucil has been linked to PML in a case report (44), and Keene et al. (2011) identified five PML cases with ibritumomab tiuxetan in WHO adverse-event databases (45). Our pharmacovigilance findings provide supportive, population-level signal evidence. In addition, mycophenolate mofetil (MMF; ROR 12.75, PRR 12.70) and azathioprine (ROR 14.65, PRR 14.57) also carry boxed warnings for PML. These observations reflect real-world pharmacovigilance data across heterogeneous underlying disorders; differences in observed PML risk are influenced by treatment duration and intensity, underlying disease, and latency from drug initiation to PML onset (4, 5, 25).

Natalizumab is indicated as monotherapy for relapsing forms of MS. Concerns about PML led to marketing suspension in 2005, followed by reintroduction with a risk-management plan (TOUCH program) and an FDA boxed warning (29). Three established risk factors for natalizumab-associated PML have been identified (5, 46): treatment duration >24 months, prior immunosuppressant use, and anti–JCV antibody positivity. Accordingly, risk is lowest in seronegative individuals (~1/10,000) and highest (~1/100) in those treated for >5 years with Stratify-JCV index values >1.5 (5). PML does not occur uniformly among patients with MS on natalizumab for 2 or more years and JCV antibody-positive, suggesting that other risk-modifying factors are at play. Hatchwell et al. performed a case–control analysis that matched patients with high PML risks who did not develop PML (47). The study demonstrated that four gene variants from those with natalizumab-related PML are associated with the risk of drug-related PML. The gene test of risk for PML would be helpful in identifying a subpopulation at particularly high risk for its occurrence when being treated with natalizumab (48).

All anti-CD20 mAbs evaluated showed PML signals in our analysis—rituximab (ROR 41.8, PRR 41.29), obinutuzumab (ROR 14.75, PRR 14.68), ofatumumab (ROR 3.06, PRR 3.05), and ocrelizumab (ROR 3.05, PRR 3.05)—and all carry PML warnings; rituximab and obinutuzumab have boxed warnings. Epcoritamab (ROR 11.15, PRR 11.11), a CD3×CD20 bispecific T-cell engager distinct from anti-CD20 mAbs, currently does not carry a PML warning in its SPC. Since 1997, rituximab has gained approvals across multiple indications (non-Hodgkin lymphoma, rheumatoid arthropathies [RA], CLL, granulomatosis with polyangiitis, microscopic polyangiitis, and pemphigus vulgaris) and induces prolonged B-cell depletion (1). Baseline PML risk varies by disease, being highest in hematologic malignancies, intermediate in granulomatosis with polyangiitis/microscopic polyangiitis, and lowest in RA (1). In hematological patients undergoing rituximab therapy, possibly in a synergistic manner, both the drug and the disease itself may act as a strong immunosuppressive stimulus. In our study, rituximab-associated PML had a median TTO of 14.1 months. From the Southern Network on Adverse Reactions, the median time from the first rituximab dose to PML diagnosis was 15.0 month (0.5–128.4), of which the majority of patients had received rituximab for hematological malignancies (non-Hodgkin lymphoma [59%] and CLL (65 [28%]) (1). Another reported median TTO in rituximab-treated non-transplant patients with B-cell malignancy or autoimmune disease without HIV was reported as 16.0 months (range, 1.0-90.0 months) (30). In cases where the onset of PML was soon after the start of therapy, the underlying condition or previous drug therapy may have been the cause of PML, rather than the new drug. Concurrent drug analysis of PML cases associated with rituximab in the treatment of hematological malignancies identified potential synergistic effects, particularly with fludarabine and bendamustine (1). These findings support the elevated PML risk associated with bendamustine and fludarabine. Since antineoplastic drugs are frequently administered in combination, it is difficult to identify the specific agent responsible for PML risk, particularly in patients with underlying immunosuppressive conditions.

Among cytotoxic chemotherapeutic agents, bendamustine and fludarabine currently carry PML warnings in their prescribing information. The EMA SPC for methotrexate includes PML in its risk warnings, whereas the FDA label does not list this ADR at present. For other cytotoxic antineoplastics—doxorubicin, cyclophosphamide, busulfan, etoposide, vincristine, melphalan, azacitidine, chlorambucil, epirubicin, thiotepa, mitoxantrone, hydroxycarbamide, and idarubicin—PML is not recognized in the SPC. Our data nonetheless showed disproportionality signals involving these agents. These drugs are widely used for neoplastic diseases, particularly hematologic malignancies; cyclophosphamide is also used for immune-mediated rheumatic disorders and for DLBCL and acute lymphoblastic leukemia. In VigiBase, Melis et al. (2015) (32) reported signals for doxorubicin, cyclophosphamide, busulfan, etoposide, vincristine, melphalan, chlorambucil, thiotepa, and mitoxantrone. Given the combined effects of underlying oncologic disease and treatment-induced immunosuppression, PML should be considered in the differential diagnosis when unexplained neurological symptoms arise (e.g., cognitive decline, motor deficits, visual disturbance).

Glucocorticoids are frequently combined with other immunosuppressants for treatment of immune-mediated/autoimmune diseases or with antineoplastic drugs for treatment of hematological malignancies. Glucocorticoid monotherapy and combination regimens have both been associated with PML development in case reports. An 80-year-old woman with ANCA-associated renal vasculitis developed PML after 6 months of prednisolone alone (49). In this case, the patient was only using steroids, but other factors such as advanced age, ANCA-associated renal vasculitis, and renal failure may have contributed to a more markedly immunosuppressed state. Thus, these factors might have contributed to the etiology of PML. In a review by Calabrese et al., 22 patients with SLE developed PML: one received cyclosporine (boxed PML warning), eight received low-dose prednisone (<15 mg/day) and/or an antimalarial, and one had no recent immunosuppression; in earlier treatment histories, one had MMF (boxed warning) and three had cyclosporine (50). Thus, while our study identified prednisolone, prednisone, dexamethasone, and methylprednisolone as signal-bearing drugs, clinical appraisal must account for patient susceptibility and concomitant therapies.

As for leflunomide, sirolimus, antithymocyte globulin (ATG), and basiliximab, although PML is not listed in the prescribing information for these immunosuppressants, our pharmacovigilance study identified PML risk signals through disproportionality analysis. Leflunomide is an immunosuppressive agent primarily indicated for RA, with limited use in systemic LE in select cases. A 68-year-old man was diagnosed with PML after his RA regimen was changed from azathioprine to leflunomide, with therapy duration not reported (51). McCalmont et al. (52) documented a fatal suspected PML case in a cardiac transplant recipient who had received sirolimus maintenance therapy for two years. The initial post-transplant immunosuppressive regimen consisted of triple therapy with cyclosporine, MMF (boxed warning), and prednisone. At 6 months post-transplantation, cyclosporine was substituted with sirolimus due to nephrotoxicity. A case of ATG-related PML has also been reported in the literature. The patient was a 15-year-old male who received ATG and rituximab conditioning therapy before haploidentical hematopoietic stem-cell transplantation without post-transplant graft-versus-host disease prophylaxis; he subsequently developed PML. The condition was successfully managed with donor-derived virus-specific T-cell infusions (53). Klintmalm et al. (54) reported a fatal PML case in a 51-year-old male liver transplant recipient 6 months post-operation. The patient received basiliximab induction (20 mg × 2 doses), maintenance immunosuppression with belatacept (high dose), and MMF (3–4 g/day for 7.5 weeks vs. the recommended 2 g/day). Another fatal PML case was reported in a 63-year-old White woman who received an extended criteria donor kidney. Her immunosuppressive protocol comprised basiliximab, belatacept, MMF (boxed warning for PML), and corticosteroids (55). In the above cases, possibly in a synergistic manner, both the drugs and the underlying disease may act as a strong immunosuppressive stimulus. Signal-detection analysis in the WHO adverse drug reaction database identified leflunomide, ATG, and basiliximab as drugs associated with PML signals (32). Early recognition and treatment are key to survival. JCV-related PML should be considered in any transplant recipient who develops neurological features suggestive of PML, regardless of the immunosuppression regimen.

The pharmacovigilance analysis also revealed a significant association between hydroxychloroquine and PML. This risk is not currently specified in the FDA-approved prescribing information. Several related case reports have been published in connective tissue disease, systemic lupus, and RA (50, 56, 57). In a case report by Scabini et al. (56), a patient with mixed connective tissue disease developed PML and died two months after hospital admission, despite receiving only minimally immunosuppressive hydroxychloroquine therapy (200 mg/day for 5 years) and low-dose corticosteroids (withdrawn after 3 years). In another case report, a 66-year-old male with a 4-year history of questionable RA developed PML while on maintenance therapy with hydroxychloroquine and prednisone (titrated up to 40 mg/day during bouts of increased stiffness and swelling). The patient died approximately one month after PML diagnosis (57). Given these findings from both population-level signal detection and individual case evidence, clinicians should exercise heightened vigilance for potential hydroxychloroquine-related PML, particularly in patients with underlying autoimmune conditions.

In pharmacovigilance assessments, the temporal relationship between drug exposure and the TTO of serious ADRs is crucial. Elucidating ADR profiles enables (1) identification of patients at risk, (2) determination of specific risk periods during treatment, and (3) implementation of targeted prevention strategies or early diagnostic interventions. In our pharmacovigilance investigation, we evaluated predisposing conditions associated with drug-related PML, focusing on hematological malignancies, autoimmune diseases, and post-transplant immunosuppressive states. Our data show that PML occurs more commonly in MS (composition ratio 32.28%) and B-cell non-Hodgkin lymphomas (composition ratio 9.44%).

We further analyzed the TTO of different drug-related PML using cases with TTO > 60 days. The reported TTO is a proxy for the actual TTO of the adverse reaction (18), and rare adverse effects like PML may be diagnosed late because of nonspecific early symptoms and neuroimaging challenges. The calculated median TTO from initiation of therapy to PML diagnosis was 13.6 months (95% CI: 11.5–15.9) for antineoplastic drugs and 42.4 months (95% CI: 39.7–44.1) for non-antineoplastic drugs. Log-rank tests revealed significant differences in PML incidence between these groups. Maas et al. found a mean time to asymptomatic lesions (only in natalizumab-induced PML) of 36.4 months (95% CI 27.0–45.9), while the mean time from drug initiation to first symptoms was significantly shorter in the neoplasm group [mean 14.2 months (95% CI 9.3–19.2)] than in other disease categories (33). Our findings support that TTO in patients with malignancy, especially those with hematological malignancy, is shorter than in patients with other diseases. Natalizumab-related PML (case breakdown: MS [571], Crohn’s disease [5], unknown [6]) had a median TTO of 43.9 months (42.0–46.6). This closely aligns with the 1,463-day median TTO reported by Oshima et al. for MS patients (28). Clinicians should remain alert to the possibility of PML regardless of treatment duration; in patients receiving immunosuppressive agents, new CNS symptoms should prompt suspicion of PML.

Our study has some limitations. Disproportionality analyses of spontaneous reporting systems can detect signals of rare ADRs not observed in premarketing clinical studies—owing to strict inclusion criteria, small sample sizes, and short trial durations—thus providing valuable hypothesis-generating insights but with inherent constraints. Duplicate reports may persist if manufacturers submit the same case, potentially skewing counts toward drugs frequently used in combination, including in refractory musculoskeletal and connective tissue disorders; this makes the natalizumab data particularly notable given its indication as monotherapy. To mitigate this, we applied sequential exclusions based on patient identifiers. Absolute case numbers may also be influenced by time on the market and exposure size. Additionally, reporting within specific active pharmacovigilance initiatives—such as REMS for natalizumab and rituximab (RADAR) and disease activity registries—may lead to higher reporting rates. Alatawi et al. (58) found substantial under-reporting of most ADE pairs in FAERS, with exceptions such as etanercept–malignancy and lamotrigine–dermatological ADEs; biological agents and drugs with a narrow therapeutic index showed relatively higher reporting rates. Similar reporting bias may affect our derived dataset for natalizumab and rituximab. Finally, as a spontaneous reporting system, FAERS lacks sufficient detail to confirm PML diagnoses according to the criteria of Berger et al. (59). Further investigation is needed to identify risk factors for JCV reactivation leading to PML during treatment with these immunomodulating therapies.

## 5 Conclusion

We identified 28 drugs without prior PML associations in regulatory labeling, spanning antineoplastic agents, immunosuppressants, glucocorticoids, antimalarials, and CAR-T therapies. Notably, isatuximab emerged as newly associated with PML, with no previous reports in regulatory documents or the published literature. Novel disproportionality signals were detected for three mAbs, including daratumumab, elotuzumab, and epcoritamab, which also lack prior mentions of PML in their labels.

Natalizumab and rituximab exhibited significantly higher PML risks than other immunomodulators, consistent with the higher frequency of PML in patients with MS and B-cell non-Hodgkin lymphomas. Additionally, we confirmed the median TTOs for natalizumab, rituximab, fingolimod, cyclophosphamide, MMF, fludarabine, tacrolimus, obinutuzumab, dimethyl fumarate, and bendamustine. The median TTO for antineoplastic drugs was 13.6 months (95% CI: 11.5–15.9), significantly shorter than for non-antineoplastic drugs, 42.4 months (95% CI: 39.7–44.1).

These findings underscore the need for vigilant monitoring for PML throughout drug therapy. The newly identified signals warrant dedicated epidemiological studies to confirm causality and quantify absolute risks. Future research should also identify specific patient- and treatment-related risk factors to guide the safer use of these immunomodulatory therapies.

## Data Availability

The original contributions presented in the study are included in the article/supplementary material. Further inquiries can be directed to the corresponding authors.
