# Supplementary material for: Disproportionality analysis of drug-associated progressive multifocal leukoencephalopathy: roles of underlying diseases and immunomodulatory therapies in FAERS
Source: Front Immunol. 2026 Jan 2;16:1707211. doi: 10.3389/fimmu.2025.1707211 (PMC12807927; doi:10.3389/fimmu.2025.1707211)
Supplement: Supplementary file 1 [file Image1.pdf]

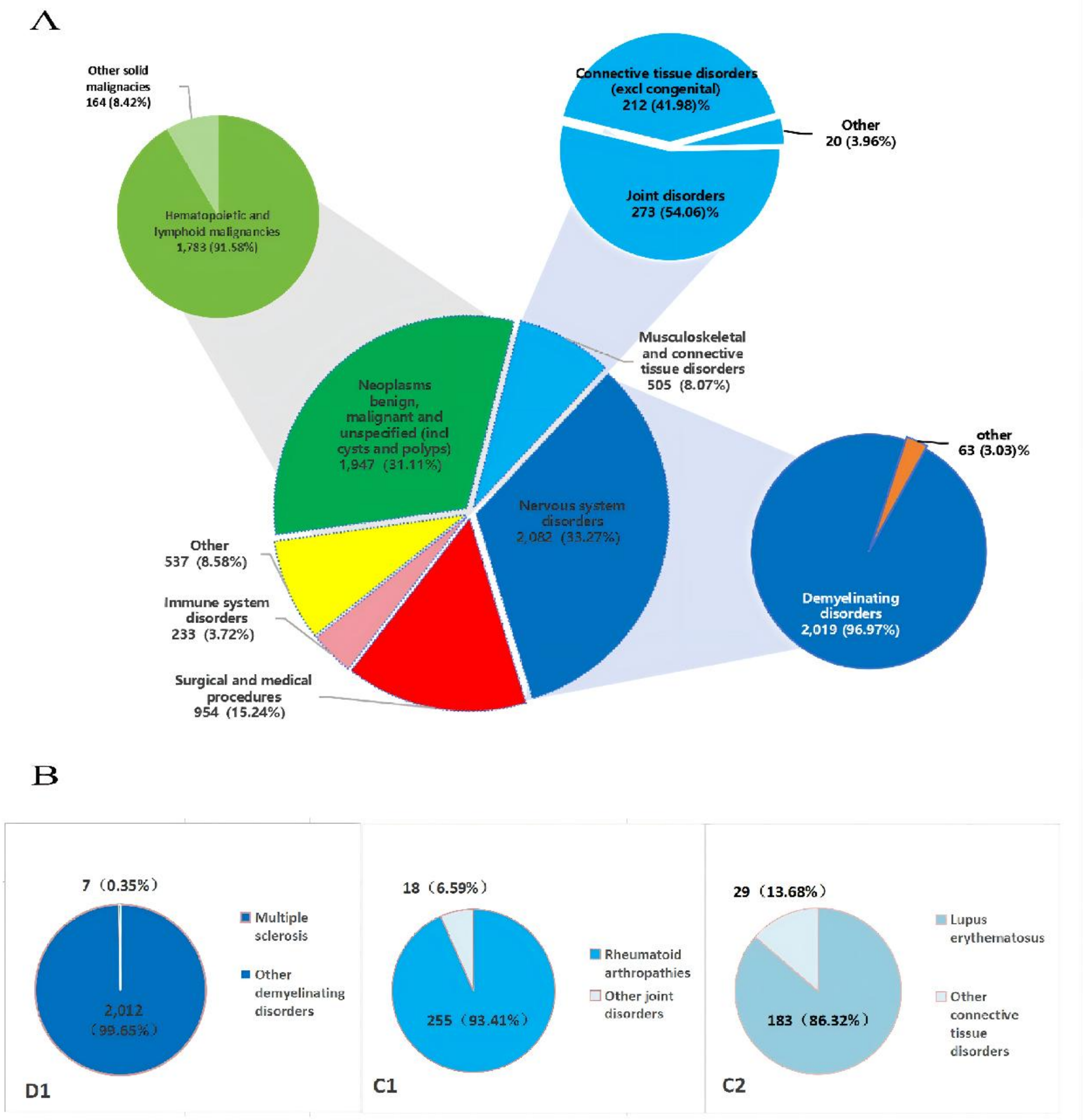

Supplementary Figure 1. Proportional distribution of drug-related PML reports by clinical reason for drug therapy (excluding cases with TTO ≤60days), FAERS (2004Q1-2024Q4). D1: Demyelinating disorders, C1: Joint disorders, C2: Connective tissue disorders (excluding congenital).
