# Supplementary material for: Disproportionality analysis of drug-associated progressive multifocal leukoencephalopathy: roles of underlying diseases and immunomodulatory therapies in FAERS
Source: Front Immunol. 2026 Jan 2;16:1707211. doi: 10.3389/fimmu.2025.1707211 (PMC12807927; doi:10.3389/fimmu.2025.1707211)
Supplement: Supplementary file 2 [file DataSheet1.docx]

**Supplementary Table 1.** Baseline characteristics of included FAERS reports (excluding cases with TTO ≤60days) (2004Q1–2024Q4)

| **Characteristics** | **Number of reports, N (%)** |
| --- | --- |
| **Sex** |  |
| Female | 3,300 (52.73%) |
| Male | 2,332 (37.26%) |
| Missing | 626 (10.00%) |
| **Age** |  |
| <18 | 80 (1.28%) |
| 18–44 | 1,167 (18.65%) |
| 45–59 | 1,665 (26.61%) |
| ≥60 | 1,915 (30.60%) |
| Missing | 1,431 (22.87%) |
| Outcomes |  |
| Death | 2,007 (32.07%) |
| Hospitalization | 1,816 (29.02%) |
| Life-threatening | 539(8.61%) |
| Disability | 60 (0.96%) |
| Required intervention to prevent permanant impairment | 3 (0.05%) |
| Others | 1,800 (28.76%) |
| Missing | 33 (0.53%) |

**Supplementary** **Table 2.** Reported drug-related PML case profiles after exclusion cases of TTO ≤60 days: Clinical reason for drug therapy from 2004Q1 to 2024Q4

| **Reason for drug therapy** | **System organ class** | **N (%)** |
| --- | --- | --- |
| Multiple sclerosis acute and progressive | Nervous system disorders | 2,012 (32.15%) |
| Hematopoietic and lymphoid malignancies | Neoplasms benign, malignant and unspecified (incl cysts and polyps) | 1,783 (28.49%) |
| Rheumatoid arthropathies | Musculoskeletal and connective tissue disorders | 255 (4.07%) |
| Immune system disorders | Immune system disorders | 233 (3.72%) |
| Lupus erythematosus (incl subtypes) | Musculoskeletal and connective tissue disorders | 183 (2.92%) |
| Other solid malignacies | Neoplasms benign, malignant and unspecified (incl cysts and polyps) | 164 (2.62%) |
| Immunosuppressant drug therapy | Surgical and medical procedures | 187 (2.99%) |
| Other reason for drug therapy | * | 1,441 (23.03%) |

*Incl* including. * involving multiple system organ classes or with missing data.

**Supplementary Table 3.** Sub-classification of hematopoietic and lymphoid malignancies among drug-related PML cases exclusion of TTO ≤60 days: Clinical reason for drug therapy (2004Q1–2024Q4)

| Hematopoietic and lymphoid malignancies | N (%) |
| --- | --- |
| Lymphomas non-Hodgkin's B-cell | 593 (9.48%) |
| Lymphomas non-Hodgkin's T-cell | 26 (0.42%) |
| Lymphomas non-Hodgkin's unspecified histology | 236 (3.77%) |
| Lymphomas Hodgkin's disease | 51 (0.81%) |
| Lymphomas NEC | 75 (1.20%) |
| Leukemias | 496 (7.93%) |
| Plasma cell neoplasms | 276 (4.41%) |
| Hematopoietic neoplasms  (excl leukemias and lymphomas) | 30 (0.48%) |
| Total | 1,783 (28.49%) |

*NEC*: Not Elsewhere Classifiable, *excl* excluding.

**Supplementary Table 4.** Top 30 drugs with the highest signal intensity according to the ROR based on FAERS (2004Q1-2024Q4).

| **Drug name** | **Before excluding TTO ≤60 days** | |  | **Drug name** | **excluding TTO ≤60 day** | |
| --- | --- | --- | --- | --- | --- | --- |
|  | **N** | **ROR rank** |  |  | **N** | **ROR ranks** |
| chlorambucil | 16 | 42.39 | chlorambucil | | 16 | 46.51 |
| rituximab | 1,296 | 41.80 | ibritumomab tiuxetan | | 14 | 45.75 |
| ibritumomab tiuxetan | 14 | 41.71 | rituximab | | 1,217 | 43.35 |
| natalizumab | 1,848 | 40.70 | natalizumab | | 1,670 | 40.21 |
| bendamustine | 101 | 29.42 | fludarabine | | 76 | 28.77 |
| fludarabine | 84 | 29.00 | bendamustine | | 89 | 28.42 |
| stavudine | 3 | 22.21 | belatacept | | 16 | 18.89 |
| brentuximab vedotin | 47 | 17.90 | busulfan | | 35 | 16.89 |
| belatacept | 16 | 17.22 | brentuximab vedotin | | 40 | 16.70 |
| busulfan | 35 | 15.39 | azathioprine | | 32 | 16.07 |
| obinutuzumab | 42 | 14.75 | thiotepa | | 8 | 15.83 |
| azathioprine | 32 | 14.65 | obinutuzumab | | 39 | 15.02 |
| thiotepa | 8 | 14.43 | efalizumab | | 13 | 14.88 |
| efalizumab | 13 | 13.56 | mycophenolate mofetil | | 255 | 13.18 |
| mycophenolic acid | 271 | 12.75 | prednisolone | | 137 | 12.40 |
| prednisolone | 143 | 11.79 | teclistamab | | 7 | 11.83 |
| doxorubicin | 132 | 11.21 | alemtuzumab | | 42 | 11.12 |
| epcoritamab | 4 | 11.15 | melphalan | | 23 | 10.63 |
| cyclophosphamide | 120 | 11.06 | cyclophosphamide | | 105 | 10.60 |
| teclistamab | 7 | 10.78 | doxorubicin | | 113 | 10.51 |
| alemtuzumab | 44 | 10.62 | idarubicin | | 4 | 10.35 |
| polatuzumab vedotin | 9 | 10.49 | prednisone | | 112 | 9.57 |
| melphalan | 24 | 10.11 | hydroxychloroquine | | 51 | 9.43 |
| idarubicin | 4 | 9.44 | epcoritamab | | 3 | 9.17 |
| mitoxantrone | 6 | 8.89 | vincristine | | 23 | 9.11 |
| prednisone | 113 | 8.79 | basiliximab | | 5 | 9.00 |
| vincristine | 24 | 8.67 | polatuzumab vedotin | | 7 | 8.95 |
| hydroxychloroquine | 51 | 8.59 | methylprednisolone | | 73 | 8.69 |
| fingolimod | 245 | 8.28 | fingolimod | | 232 | 8.61 |
| basiliximab | 5 | 8.21 | elotuzumab | | 5 | 8.54 |

**Supplementary Table 5.** Event year of natalizumab-associated PML reports (2015, FAERS)

| Event_year | Number of reports |
| --- | --- |
| 2007 | 3 |
| 2008 | 4 |
| 2009 | 7 |
| 2010 | 20 |
| 2011 | 33 |
| 2012 | 49 |
| 2013 | 73 |
| 2014 | 99 |
| 2015 | 87 |
| missing | 83 |
| Total | 458 |

**Supplementary Table 6.** Time to onset analysis of Progressive Multifocal Leukoencephalopathy for implicated drugs (2004Q1-2024Q4)

|  | **Drug name** | **N** | **Interquartile range**  **(mon)** | **Median**  **(mon)** | **Minimum**  **(mon)** | **Maximum**  **(mon)** |
| --- | --- | --- | --- | --- | --- | --- |
| 11 | bortezomib | 10 | 3.2–35.1 | 8.1 | 2.7 | 50.0 |
| 12 | cyclosporine | 8 | 9.1–67.5 | 20.1 | 2.9 | 82.0 |
| 13 | brentuximab vedotin | 8 | 3.5–17.8 | 7.7 | 2.0 | 44.5 |
| 14 | ofatumumab | 8 | 8.0–29.0 | 17.0 | 2.2 | 46.8 |
| 15 | alemtuzumab | 7 | 2.2–8.0 | 5.0 | 2.1 | 13.6 |
| 16 | axicabtagene ciloleucel | 7 | 7.5–21.1 | 14.1 | 7.3 | 24.6 |
| 17 | daratumumab | 6 | 4.8–18.5 | 11.1 | 2.7 | 24.6 |
| 18 | doxorubicin | 6 | 4.3–24.0 | 11.3 | 4.2 | 39.0 |
| 19 | methotrexate | 6 | 46.2–168.9 | 143.0 | 4.8 | 200.2 |

*N* number, *m* month, one year = 365.25 days.
